# Larks under pressure: The genetic background of the morning chronotype may contribute to depression in interaction with stress

**DOI:** 10.1192/j.eurpsy.2024.1117

**Published:** 2024-08-27

**Authors:** M. Csikos, S. Krause, Z. Gal, G. Bagdy, G. Juhasz, X. Gonda, D. Torok

**Affiliations:** ^1^Department of Pharmacodynamics; ^2^ NAP3.0-SE Neuropsychopharmacology Research Group, Hungarian Brain Research Program; ^3^Department of Psychiatry and Psychotherapy, Semmelweis University, Budapest, Hungary

## Abstract

**Introduction:**

Depression is a highly prevalent, multifactorial, complex disorder, its etiology is assumed to involve both genetic and environmental factors. Genetic factors, including biological clock genes such as *CLOCK* and *SIRT1*, have been linked to depression, particularly its symptom related sleep disturbances. Environmental factors also play a crucial role in the background of depression, particularly in interaction with genetic factors. Known environmental stress factors include stress caused negative life events or childhood adversities.

**Objectives:**

This study aims to delve into the chronotype-specific impacts of genes previously correlated with circadian functionality on the pathomechanism of depression in interaction with environmental stress factors.

**Methods:**

A genome-wide association study on the ‘morning chronotype’ phenotype was conducted with Plink2, utilizing data from the UK Biobank discovery sample (N = 139135). Using LDPred2we derived a polygenic risk score (PRS) for the NewMood Hungarian dataset (N = 1820). We performed pathway-specific analyses including genes implicated within the genetic pathway, drawing on prior research findings. Specifically, we selected the top genes (with a false discovery rate-corrected p-value < 0.05) from the “responders vs. non-responders” analysis conducted by Jerome C. Foo et al.Transl Psychiatry 2019; 9 343). We performed a main effect analysis investigating the pathway specific PRS’s effect on BSI depression scores and interaction analyses using life course (number of negative life events in the past life) and recent (number of negative life events in the past year) stress scores to investigate how the interaction term predicts depression in our target sample.

**Results:**

Our primary analysis revealed a nominally significant protective effect (beta = -20.90938, p = 0.070218). Subsequently, in the context of our interaction analysis, we identified significant risk associations, both with lifetime stress (beta = 13.7416, p = 0.0171) and recent stress (beta = 24.6034, p = 0.0038)

**Conclusions:**

Our study unveiled a protective role in our primary analysis, juxtaposed with risk associations in our interaction analyses. This intriguing dichotomy underscores that this genetic pathway, associated with circadian dysregulation, exerts a protective influence in association with the morning chronotype. However, it transitions into a predisposing factor for depression when influenced by environmental stress factors.

Considering these findings, our study substantiates the hypothesis that both circadian genes and chronotype contribute to the pathogenesis and clinical manifestation of depression. Additionally, it underscores the pivotal role of stress as a contributing factor in the intricate pathogenesis of depression.

**Disclosure of Interest:**

None Declared

